# What about males? Exploring sex differences in the relationship between emotion difficulties and eating disorders

**DOI:** 10.1186/s40337-022-00715-6

**Published:** 2022-12-13

**Authors:** L. Vuillier, J. Joseph, M. Greville-Harris, L. May, M. P. Somerville, A. Harrison, R. L. Moseley

**Affiliations:** 1 Department of Psychology, Bournemouth University, Poole, UK; 2 Dorset Healthcare University NHS Foundation Trust, Poole, UK; 3 UCL Institute of Education, University College London, London, UK

## Abstract

**Objective:**

While eating disorders (EDs) are more commonly diagnosed in females, there is growing awareness that men also experience EDs and may do so in a different way. Difficulties with emotion processing and emotion regulation are believed to be important in EDs, but as studies have involved predominantly female samples, it is unclear whether this is also true for males.

**Methods:**

In a sample of 1604 participants (n = 631 males), we assessed emotion processing and emotion regulation in males with EDs (n = 109) and compared results to both females with EDs (n = 220) and males from the general population (n = 522). We also looked at whether emotion processing and emotion regulation difficulties predicted various aspects of eating psychopathology and whether this was moderated by sex. We assessed emotion processing with the Toronto Alexithymia Scale, emotion regulation with the Difficulties in Emotion Regulation Scale and the Emotion Regulation Questionnaire, and eating psychopathology with the Eating Disorder Examination Questionnaire.

**Results:**

We found that males with ED, like their female counterparts, suffered from emotion processing and emotion regulation deficits. We did find some sex differences, in that males with EDs tended to report more difficulties with their emotions as well as a more externally oriented thinking style compared to females with EDs. Difficulties with emotion processing and emotion regulation were strongly predictive of various aspects of eating psychopathology in both sexes. Importantly, we found that sex moderated the relationship between cognitive reappraisal and eating restraint. As such, low use of reappraisal was found to be associated with higher levels of restraint in females but not in males.

**Discussion:**

Difficulties with emotion processing and emotion regulation are associated with eating psychopathology in both males and females. Reappraisal was not found to be associated with reduced eating psychopathology in males, suggesting a cautious approach to interventions targeting this strategy. Research around explanatory mechanisms and interventions must adopt a broader viewpoint including those that are traditionally overlooked in EDs.

**Supplementary Information:**

The online version contains supplementary material available at 10.1186/s40337-022-00715-6.

## Plain English summary

While eating disorders (EDs) are more common in females, males also suffer from these conditions and are generally neglected in research around EDs. Difficulty identifying and managing emotions is believed to be important in the development and maintenance of EDs, but as studies have been conducted mostly in females, it is unclear whether this is also true for males. We recruited 1604 participants (631 were males, and 329 were diagnosed with EDs comprising 109 males and 220 females) and compared how males and females processed and regulated their emotions. We found that males with EDs, like their female counterparts, suffered from difficulties identifying and regulating their emotions, though they showed a slightly different profile of difficulties. While difficulties with emotions were associated with ED behaviours in both sexes, difficulties using reappraisal, an emotion regulation strategy where one reinterprets an event from a different perspective, were associated with restraint in females but not in males. This suggests that while interventions to help with emotional functioning could be beneficial for both women and men with EDs, the different emotional profiles of men with EDs must be considered, as interventions targeting particular emotional processes (e.g. reappraisal) may be relevant for women but not men.

**Supplementary Information:**

The online version contains supplementary material available at 10.1186/s40337-022-00715-6.

## Background

Though eating disorders (EDs) are more common in females [1], incidence in males is rising [2]. Problems with emotional functioning are known to play a role in the maintenance and development of EDs [3–5], and from this knowledge treatments for EDs, such as Dialectical Behaviour Therapy for EDs [6] have been developed. However, few studies have explored the emotional difficulties of males with EDs. This study aims to build upon the preliminary findings of previous research [7] and increase our understanding of male EDs by exploring emotional functioning in males and females with EDs.

It is now widely agreed that EDs are characterised by problems across the emotion cycle. For example, people with EDs commonly exhibit a lack of awareness and recognition of their emotional states and an inability to clearly define and label them, a state known as alexithymia [4, 5, 8]. Alexithymia is characterised by deficits in the earliest stages of emotion processing. It includes atypical attention to emotion-related stimuli and subsequently affects conscious appraisal of emotions and behavioural responses later on in the cycle [9, 10]. The ability to anticipate, recognise and differentiate between emotions may also be a prerequisite for identifying and flexibly utilising adaptive regulation strategies [11–13]. As such, a relationship has indeed been demonstrated between alexithymia and emotion regulation deficits in anorexia nervosa (AN) [14], binge eating disorder (BED) [15] and bulimia nervosa (BN) [16]. People with EDs also struggle to flexibly employ emotion regulation strategies in a context-dependent manner [17], both under-utilising adaptive strategies (like reappraisal and problem-solving) and over-relying on maladaptive ones [4, 18]. Of these, non-acceptance of emotions is most strongly associated with ED psychopathology [19] and may underpin other maladaptive forms of emotion regulation seen in people with EDs, such as avoidance or suppression [20]. While there is strong evidence for a transdiagnostic role of emotion processing and regulation deficits in the maintenance of ED psychopathology [21], the centrality of these difficulties for EDs is further solidified by emerging evidence that stalled development of emotion regulation strategies precedes and elevates risks of ED pathology in adolescence [22].

While this body of research has informed commonly-used psychological interventions such as Dialectical Behavioural Therapy [23, 24], the generalisability and hence clinical utility of these findings is hampered by a recurrent issue in ED research: the lack of representation of male participants in studies [25]. Given that EDs occur at a lifetime prevalence of 0.74% in people assigned male at birth [26], it is troubling that current knowledge around causal mechanisms and optimal treatment for EDs may be unrepresentative and less (if at all) relevant for a considerable swathe of the entire ED population. While this has not been ascertained, it is reasonable to assume that emotion processing and regulation processes may differ in males and females with EDs. In cisgender men and women,^1^ sex differences are evident in the functioning and connectivity of neural circuitry underpinning emotion processing and regulation [28–30]. Behaviourally, these effects emerge early on in the emotion cycle, with women tending towards greater emotional awareness [31], and men towards greater difficulty in identifying and describing their emotions and an inclination towards the more externally-orientated thinking style characteristic (at high levels) of alexithymia [32, 33]. Men and women also differ in their reported use of emotion regulation strategies [31], most markedly in expressive suppression being reported in men [34–36], who may exhibit greater skill at it [37], and more use of reappraisal by females [38]. There is some suggestion that women tend towards a greater repertoire of other maladaptive strategies [36, 39], though this may be a reflection of their tendency to report more usage of nearly all types of emotion regulation strategy [31], and variability in findings may derive from effects of age [40]. Of particular relevance in this context, however, are findings that sex also moderates the relationships between emotion processing and regulation deficits and mental and physical health complaints [31, 35, 41, 42]. While there is a robust case that emotion processing and regulation deficits might contribute to ED psychopathology in women, this cannot be assumed to be the case in men.

To address this gap, two lines of research enquiry may be relevant. The first line of enquiry concerns whether differences in emotional functioning are even *present* in men with EDs. Comparisons between men with and without EDs are informative as to whether emotion processing and regulation deficits distinguish males with EDs from those without. Secondly, comparisons between men and women with EDs help to illustrate whether emotion processing and regulation deficits are a feature of EDs in both groups and whether this profile differs according to sex. The only study to consider both comparisons, to our knowledge, is that of Agüera et al. [7]. In comparison with healthy male controls (n = 78), these authors found that males with EDs (n = 62) experienced more difficulties with all facets of emotion regulation represented in the Difficulties in Emotion Regulation Scales (DERS) [43]. In the comparison of male and female (n = 656) individuals with EDs, they found similar difficulties with emotional awareness, but greater problems in females with accepting their emotional responses, accessing emotion regulation strategies, having emotional clarity, inhibiting impulsive behaviour and engaging in goal-directed behaviour during negative emotional experiences. While this study provides an important initial foray into profiling the emotional difficulties of men with EDs, the DERS does not sufficiently distinguish between difficulties associated with early and late stages in the emotion cycle. Two of its subscales, ‘clarity’ and ‘emotional awareness’, appear to partially overlap with the alexithymia construct as measured by the Toronto Alexithymia Scale, albeit *also* loading on emotion regulation processes assumed to occur at later stages of processing [16, 44]. Alexithymia is, however, a broader construct which *precedes* emotion regulation deficits [11]; its effects are proposed to occur at the earliest stages of the emotion life-cycle in the encoding, processing and accessing of emotional information, such that this feature effectively restricts an individual’s ability to utilise emotional information, and to clearly differentiate between emotions, in later processing stages [45]. As such, it is still unclear to what extent men with EDs experience alexithymia in comparison with their female counterparts and healthy male controls. An additional limitation of this study is that while it focuses on *difficulties* in emotion regulation, the authors did not examine differential strategies for emotion regulation in men and women with EDs, as might be expected on the basis of findings from the general population. As such, comparisons between males with EDs and relevant counterparts are few and do not fully address gaps in present knowledge.

While it is important to examine the presence of emotion processing and regulation differences in men with EDs, a second and arguably more important line of enquiry is to look at how emotion processing and regulation deficits are *associated* with ED psychopathology in males and females. Indeed, the greater importance of such studies, over group comparisons, lies in the possibility that differences in emotion processing and regulation might exist but be irrelevant to—or not associated with—ED psychopathology in men. Unfortunately, literature in this area is also somewhat inconclusive. General difficulties with emotion regulation have been associated with certain aspects of ED psychopathology in both men and women: these include general eating psychopathology [46], binge-eating [47–49], and concerns about weight, shape and body dissatisfaction [50–52], although the latter seems more focused on muscularity in males. Eating restraint has been associated with emotion regulation difficulties in females [53], but their relationships to emotion processing and regulation in males are, to our knowledge, conflicting. While some studies suggest that this relationship exists unmoderated by sex [49], others failed to find a relationship between emotion regulation difficulties and restricting behaviours in male samples [47]. In addition to potentially moderating associations between general emotion regulation difficulties and eating psychopathology, sex may moderate associations between ED psychopathology and specific emotion regulation strategies, as well as emotion processing deficits. For instance, some studies suggest that the negative correlation between the use of adaptive emotion regulation strategies (such as reappraisal) and eating pathology is stronger in females than in males, but that the association between maladaptive strategies (e.g. suppression) and ED behaviour is not moderated by sex [4]. While deficits in emotion processing, such as alexithymia, have also been robustly linked to eating psychopathology in predominantly female samples [5, 54, 55], very few have examined associations between alexithymia and ED psychopathology in males. Two studies suggest that associations between alexithymia, bulimia and “emotional eating” might be stronger in males than females [56, 57], but both involved very small male samples. Overall, while several of these studies have examined men specifically [47, 51, 52], few have been able to convincingly examine moderating effects of sex within sizeable samples of men and women with EDs, meaning that it is still unclear whether emotion processing and regulation difficulties have the same psychopathological relevance to EDs in men. Statistical moderation effects however translate to clear clinical importance: interventions developed to alleviate ED symptoms via targeting emotion processing and regulation difficulties [58], for instance, will be less relevant and less efficacious for males if the difficulties in question are not associated with eating psychopathology in people of this sex.

The present study aimed to examine both the *presence* and the *relevance* of emotion processing and regulation deficits in males with EDs in a large sample of 1604 participants (n = 631 males, n = 973 females) with and without a diagnosed ED (n = 329 and n = 1275 respectively). Employing both comparative and moderation analyses, our goals and expectations were as follows:

Firstly, we aimed to clarify the emotional functioning of men with EDs in relation to healthy male controls, extending previous investigations of emotion regulation difficulties [7] by including additional, more well-defined measures of specific emotion regulation strategies [59] and early emotion processing difficulties as reflected by alexithymia. Based on the Agüera study [7] and the perspective that dysfunctional emotion processing and regulation is a transdiagnostic component of EDs [21, 60, 61], we hypothesised that men with EDs would exhibit deficits across all measures of emotion processing and emotion regulation.

Secondly, we aimed to examine the emotional functioning of men with EDs in comparison with their female ED counterparts. While sex differences were broadly expected across all emotion processing and regulation measures based on literature from the general population [31, 32, 38], the same studies indicate that if typical norms are adhered to, we might particularly expect to see higher alexithymia and greater use of suppression in men with EDs, and greater use of reappraisal in women with EDs.

Thirdly, we aimed to clarify the relevance of emotion processing and regulation deficits to EDs in men and women. Given previous inconsistencies in the literature, we had no a priori hypotheses for this exploratory analysis, over and above predicting relationships between emotion processing, emotion regulation and ED psychopathology. By examining moderating effects of sex on these same associations, we hoped to elucidate if different aspects of emotion processing and regulation dysfunction are of particular clinical relevance for men and women with EDs.

## Methods

### Participants

#### Participant recruitment occurred via two streams

The first dataset consisted of 905 participants (n = 255 males, n = 643 females, n = 7 non-binary; n = 67 with an ED) recruited in 2019 from a community sample. Of the sample, 206 participants were students recruited from Bournemouth University, and the other participants (n = 699) were recruited through social media (Facebook and Reddit).

The second dataset consisted of 707 UK participants (n = 376 males, n = 330 females, n = 1 non-binary; n = 265 with an ED) who were recruited in July 2020 on Prolific to complement the first sample. We recruited these participants to increase both our male sample and the number of participants with an ED. These participants completed a screening survey and participants with current ED symptoms were specifically invited to participate in the full survey.

The combined dataset consisted of 1612 participants (n = 631 males, n = 973 females, n = 8 non-binary), for which missing data represented less than 1% of the dataset (0.58%). Because the purpose of this study was to look at sex differences, we removed the non-binary participants from the final dataset as we did not have enough participants in this category for any meaningful comparison—we however recommend future research to actively include these participants. Of our total sample (n = 1604), 329 reported that they had been formally diagnosed with an ED by a clinician, and that their eating difficulties were current (109 males, 220 females). Of these, n = 136 self-reported a diagnosis of AN (n = 35 males, n = 101 females); n = 73 of BN (n = 29 males, n = 44 females); n = 55 of BED (n = 28 males, n = 27 females); n = 29 of OSFED (Other Specified Feeding or Eating Disorder) or EDNOS (Eating Disorder Not Otherwise Specified; n = 4 males, n = 25 females); and n = 36 were categorised as ‘Other’ (e.g. some reported a dual diagnosis or did not report a specific diagnosis, n = 13 males, n = 23 females). While most of our sample (60.5%) was White British, the remainder were diverse in their ethnicity (Asian: 13.7%, European: 11%, Mixed: 9.2%, Black: 1.1%, Other: 4.4%). Mean age was 26.66 (SD = 11.17) and was not significantly different between males with EDs and females with EDs (t(321) = 0.5, *p* = 0.304), or between males with EDs and males without EDs (t(612) = 0.4, *p* = 0.343). Therefore, we did not control for age in our analyses. See Table 1 for mean age for the different subgroups and their scores on major study variables.

**Table 1:** Sample description

	Diagnosed ED (n = 329)		Non diagnosed (n = 1275)	
	Males (n = 109)	Females (n = 220)	Males (n = 522)	Females (n = 753)
Age	29.9 (9.5), 18–55	29.4 (9.5), 16–59	29.5 (11.4), 16–77	25.0 (9.6), 16–69
Illness duration	5.4 (5.8), *0–31*	8.3 (8.1), *0–40*	-	-
EDEQ				
Total	3.7 (1.1), *1.3–6* α = .91	4.2 (1.2), *0.3–6* α = .93	1.7 (1.4), *0–6* α = .93	2.5 (1.6), *0–5.9* α = .95
Restraint	3.2 (1.6), *0–6* α = .83	3.6 (1.7), *0–6* α = .84	1.4 (1.5), *0–6* α = .83	2.0 (1.8), *0–6* α = .87
Eating concerns	3.2 (1.4), *0.4–6* α = .76	3.4 (1.4), *0–6* α = .76	1.0 (1.2), *0–6* α = .83	1.5 (1.6), *0–6* α = .86
Shape concerns	4.2 (1.2), *0.8–6* α = .85	4.7 (1.3), *0.2–6* α = .89	2.2 (1.7), *0–6* α = .91	3.1 (1.8), *0–6* α = .92
Weight concerns	4.0 (1.2), *1–6* α = .76	4.5 (1.4), *0–6* α = .81	1.9 (1.6), *0–6* α = .85	2.8 (1.8), *0–6* α = .87
TAS				
Total	63.9 (11.3), *29–85*	60.2 (14.3), *0–88*	47.3 (17.3), *0–122*	52.4 (13.2), *0–86*
	α = .82	α = .86	α = .85	α = .87
DIF	23.5 (6.2), *7–35*	22.8 (6.9), *0–35*	16.1 (7.4), *0–40*	18.5 (6.7), *0–35*
	α = .87	α = .85	α = .86	α = .86
DDF	17.7 (4.1), *6–25*	17.1 (5.1), *0–25*	12.9 (6.3), *0–40*	14.7 (5.0), *0–25*
	α = .71	α = .82	α = .79	α = .81
EOT	22.7 (4.9), *11–34*	20.3 (5.2), *0–35*	18.2 (7.0), *0–42*	19.2 (4.6), *0–32*
	α = .62	α = .61	α = .55	α = .60
DERS				
Total	59.7 (12.2), *28–82*	57.6 (14.7), *0–88*	44.1 (13.5), *0–85*	46.9 (14.3), *0–88*
	α = .88	α = .91	α = .91	α = .92
Strategies	10.8 (2.9), *4–15*	10.0 (3.3), *0–15*	7.4 (3.1), *0–15*	7.7 (3.0), *0–15*
	α = .80	α = .85	α = .81	α = .86
Non-acceptance	10.5 (3.0), *3–15*	10.2 (3.6), *0–15*	7.3 (3.4), *0–15*	8.2 (3.7), *0–15*
	α = .76	α = .87	α = .84	α = .88
Impulse	9.8 (3.5), *3–15*	8.8 (3.8), *0–15*	5.9 (3.1), *0–15*	6.6 (3.4), *0–15*
	α = .87	α = .90	α = .90	α = .91
Goals	11.5 (2.6), *4–15*	11.3 (3.1), *0–15*	9.5 (3.5), *0–15*	10.4 (3.5), *0–15*
	α = .82	α = .88	α = .89	α = .91
Awareness	8.1 (2.9), *3–14*	8.5 (3.3), *0–15*	7.5 (2.8), *0–15*	7.2 (2.8), *0–15*
	α = .75	α = .84	α = .72	α = .77
Clarity	9.0 (2.9), *3–15*	8.8 (3.3), *0–15*	6.4 (3.0), *0–15*	6.8 (3.0), *0–15*
	α = .79	α = .88	α = .83	α = .87
ERQ				
Reappraisal	25.5 (7.0), *6–41*	22.7 (7.5), *0–42*	27.4 (7.1), *0–42*	27.5 (7.2), *0–42*
	α = .87	α = .87	α = .87	α = .87
Suppression	18.5 (4.7), *6–28*	16.6 (6.1), *0–28*	17.0 (5.1), *0–28*	15.1 (5.5), *0–28*
	α = .71	α = .82	α = .71	α = .75
DASS	67.5 (25.9), *0–120*	65.5 (27.0), *0–124*	38.4 (25.7), *0–126*	42.1 (27.8), *0–126*
	α = .94	α = .93	α = .94	α = .94

Mean, SD (brackets) and minimum–maximum (italicised) for each group on age, illness duration, eating psychopathology (EDEQ), alexithymia (TAS-20), emotion regulation difficulties (DERS), emotion regulation strategies (ERQ), and general distress (DASS-21). The second indented line refers to the internal consistency of each questionnaire within this sample as expressed in Cronbach’s alpha (α)

### Measures and procedure

This study received ethical approval from the Research Ethics Panels at Bournemouth University and University College London. Participants completed a number of measures which were presented through the online platform Qualtrics. Participants also answered other questionnaires not reported in the current paper, and some of the data was used in two published articles [19, 62]. The measures relevant to the present analysis as predictors, dependent variables and covariates were as follows:

### Independent variables: measures of emotion processing and regulation

#### The toronto alexithymia scale (TAS-20)

We utilised the TAS-20 [63] as an indication of deficits at the earliest stages of emotion processing. Alexithymia, the construct measured by the TAS-20, is conceptualised by the scale authors as a dimensional personality trait which affects these same early stages of emotion processing [13]. The 20 items of this short self-report measure capture three facets of alexithymia: identification of one’s own emotional states (difficulty identifying feelings: DIF), the ability to verbally describe emotional states to others (difficulty describing feelings: DDF), and an inclination away from introspection and towards externally-orientated thinking (EOT). Scores above 61 indicate a clinically substantive level of impairment. The TAS-20 has good internal consistency [64], which was confirmed in our sample (See Table 1).

#### The difficulty in emotion regulation scale, short form (DERS-SF); the emotion regulation questionnaire (ERQ)

To assess emotion regulation, we employed two measures quite different in nature. The DERS-SF [65] is an 18-item scale assessing clinical impairments in emotion regulation (as indicated by higher scores). Its items correspond to six subscales: lack of emotional clarity, lack of emotional awareness, difficulties engaging in goal-directed behaviour when upset, difficulties with impulse control when upset, non-acceptance of emotions, and limited access to emotion regulation strategies (henceforth ‘clarity’, ‘awareness’, ‘goals’, ‘impulse’, ‘non-acceptance, ‘strategies’). The ERQ [66], in contrast, is a 10-item scale which focuses on two precisely-defined emotion regulation strategies: reappraisal and suppression. The authors of both tests reported good internal consistency in their original samples; our sample, likewise, showed strong internal consistency (See Table 1).

### Dependent variable: the eating disorder examination questionnaire (EDE-Q)

Participants completed the EDE-Q [67] to assess the presence and severity of ED psychopathology. The EDEQ contains 28 questions referring to the past 28 days, with high scores indicative of severe ED psychopathology. It also has four subscales to measure various aspects of the psychopathology of EDs: restraint, eating concern, shape concern, and weight concern. The EDE-Q has strong internal consistency [68] which was confirmed in our sample (See Table 1).

### Covariate: depression, anxiety and stress scale (DASS-21)

The DASS-21 [69] is a 21-item questionnaire measuring symptoms of depression, anxiety and stress (7 items each) experienced over the past week. This scale has good internal consistency [70] which was also confirmed in our sample (Table 1). The present study used the DASS total score as a measure of general distress, indicative of both anxious and depressive symptoms [71]. Differences in these symptoms were not the focus of our analysis but were controlled for in analyses attempting to differentiate their effect from that of alexithymia (see Data analysis section).

## Data analysis

Our analysis comprised three streams, corresponding with the three goals of the study:

### Part 1: emotion differences between men with and without EDs

Aiming to elucidate the emotional functioning of men with EDs in comparison with males without EDs from the general population (henceforth ‘healthy controls’ [HC]), we hypothesised that the former group would exhibit greater impairments across all measures of emotion processing and regulation. To test this hypothesis, we performed a MANOVA comparing the two groups on 11 dependent variables (DVs): the three alexithymia subscales (DIF, DDF and EOT), the six DERS subscales (strategies, non-acceptance, impulse control, goals, awareness and clarity), and the two ERQ subscales (reappraisal and suppression); ED diagnosis being our independent variable (IV).

This analysis was performed twice in consideration of uncertainty around the centrality of alexithymia in EDs, and its causal primacy over this and other forms of psychopathology. Some researchers suggest that the appearance of alexithymia in EDs could instead be a product of general distress [72, 73]. There is some support for the effects of alexithymia on ED symptomatology being explained by anxiety and depression [74] -although see [75]. The original authors of the TAS-20 assert that the test has relative stability, reflecting personality traits that maintain consistent trends despite fluctuations in state psychopathology [70]. However, they suggest that researchers wishing to delineate effects of alexithymia from those of anxiety and depression may choose to control for these symptoms. While this may make sense from a statistical or even theoretical point of view, it does not necessarily make sense from a practical perspective given the commonality of co-occurring depression and anxiety in EDs [76, 77], and controlling for it may take away an important part of the ED pathology itself. For these reasons, we also ran a MANCOVA to control for anxiety and depression (DASS-21 Total). Both are reported in the main manuscript to reflect differences relevant to the ED group as a whole, in addition to differences specific to EDs *above and beyond* general distress.

To control for multiple comparisons, we used a Bonferroni correction, correcting the alpha level to *p* = 0.0046 to account for 11 comparisons (DVs) in each analysis.

### Part 2: emotion differences between males and females with EDs

Aiming to examine whether the profile of impairments was similar in males and females with EDs, we expected that the sex differences typically observed across men and women might be similarly reflected in individuals with EDs. While previous literature highlighted potentially greater differences in relation to emotion processing and suppression as facets of emotion regulation, these previous studies are indicative of broader sex differences, for instance in emotion regulation weaknesses (as measured by the DERS-SF). As such, we included all 11 IVs used in the previous analysis in two new MANCOVAs, comparing males and females with EDs (sex being our DV).

As previously, we performed this analysis twice, with and without controlling for general distress. In both of these analyses, however, it was necessary to consider two potential confounding variables that differed between groups. Males with EDs had lower levels of eating psychopathology (M_EDEQ_ = 3.7, SD = 1.1) compared to females with EDs (M_EDEQ_ = 4.2, SD = 1.2; t(327) = − 3.0, *p* < 0.001), and also reported a shorter illness duration (M_Males_ = 5.4, SD = 5.8, M_Females_ = 8.3, SD = 8.1, t(295) =  − 3.2, *p* < *0.0*01). As such, the analysis was performed once controlling for severity and length of illness, and once controlling for these variables in addition to general distress. Due to missing data on some of the control variables, the analysis included 98 males and 192 females. As before, we controlled for multiple comparisons using Bonferroni correction (adjusted *p* = 0.0046) in all analyses.

### Part 3: emotion difficulties as predictors of ED psychopathology

Over and above examining the presence of differences in emotion processing and regulation, our third goal was to examine the relevance of these differences for ED psychopathology. That is, we examined whether emotion processing and regulation predicted various aspects of eating psychopathology in our whole sample of participants with and without ED diagnoses, and whether sex moderated any of these effects. Given the inconsistencies in previous literature, this analysis was exploratory. First, all variables were mean-centred, and a dummy variable was created for sex (0 for males and 1 for females). Subsequently, we ran a set of 5 moderated hierarchical regressions, one for each EDEQ subscale in addition to the total. As predictors for these dependent variables, we entered sex, and all 11 emotion variables in the first block of the model, and then the interaction terms of sex with each emotion variable in the second block of the model. We ran these models again controlling for general distress. VIF scores without the interaction variables were all below 4, suggesting no issue with multicollinearity. Given this is an exploratory analysis, we did not adjust the *p* values—however, we discuss our findings with caution.

## Results

### Part 1: do males with EDs have more difficulties processing and regulating their emotions than males without EDs?

As shown in Table 2, significance tests for our individual dependent variables revealed that males with EDs reported more difficulties identifying (DIF) and describing their feelings (DDF), as well as using more externally-orientated thinking (EOT), compared to males without EDs. In relation to emotion regulation, males with EDs also reported more difficulties accessing emotion regulation strategies, more difficulties accepting emotions, more difficulties engaging in goal-directed behaviours when upset, more impulse control difficulties, less clarity about their emotions and more use of suppression compared to males without EDs. There was no difference in awareness of emotions or use of reappraisal between the two groups.

**Table 2:** Comparing ED males versus HC males, and ED males versus ED females

	Part 1:			Part 2:		
	ED males versus HC males			ED males versus ED females		
	*F*	*p*	η^2^	*F*	*p*	η^2^
*TAS*						
DIF	96.5	< .001*	.13	4.3	.039	.02
	*8.5*	.004*	*.01*	*1.2*	*.278*	*.04*
DDF	58.3	< .001*	.09	4.9	.027	.02
	*2.7*	*.103*	*.00*	*1.9*	*.172*	*.01*
EOT	39.7	< .001*	.06	18.9	< .001*	.06
	*5.3*	*.021*	*.01*	*16.1*	< .001*	*.05*
*DERS*						
Strategies	111.6	< .001*	.15	10.4	.001*	.04
	*19.6*	< .001*	*.03*	*6.3*	*.012*	*.02*
Non-acceptance	80.5	< .001*	.11	2.4	.119	.01
	*8.5*	.004*	*.01*	*0.5*	*.501*	*.00*
Impulse	132.5	< .001*	.17	10.4	.001*	.03
	*39.4*	< .001*	*.06*	*6.5*	*.011*	*.02*
Goals	29.7	< .001*	.04	1.2	.272	.00
	*0.1*	*.826*	*.00*	*0.1*	*.771*	*.00*
Awareness	4.1	.043	.01	0.00	.985	.00
	*0.4*	*.526*	*.00*	*0.0*	*.834*	*.00*
Clarity	73.8	< .001*	.11	3.7	.056	.01
	*7.5*	*.006*	*.01*	*1.2*	*.279*	*.00*
*ERQ*						
Reappraisal	6.3	.013	.01	8.9	.003*	.03
	*0.1*	*.704*	*.00*	*10.0*	.002*	*.03*
Suppression	8.3	.004*	.01	11.9	< .001*	.04
	*0.6*	*.429*	*.00*	*8.7*	.003*	*.03*

Group differences between ED males and HC males (left, Part 1) and between ED males and ED females (right, Part 2) on the three subscales of the TAS, six subscales of the DERS, and two subscales of the ERQ, also controlling for severity and length of illness (Part 2 only). The first row reports the results without controlling for general distress, the second row in italics reports results when controlling for it. *p values significant after Bonferroni correction

When controlling for general distress, we found a weaker but still statistically significant main effect of ED diagnosis on emotion processing and regulation, *F*(11, 281) = 5.5, *p* < 0.001, Wilk's Λ = 0.856, partial η^2^ = 0.14. This time, the only significantly different variables between groups were, for emotion processing, difficulties identifying emotions (DIF), and for emotion regulation, difficulties accessing emotion regulation strategies, non-acceptance of emotional states, and impulse control difficulties.

### Part 2: do males with EDs have similar difficulties processing and regulating their emotions compared to females with EDs?

There was a statistically significant main effect of sex across all our emotion processing and emotion regulation dependent variables, even after controlling for severity and length of illness, *F*(11, 282) = 4.74, *p* < 0.001, Wilk's Λ = 0.844, partial η^2^ = 0.16. Interestingly, this reflected higher scores in males with EDs across all variables. As shown in Table 2, we found that males with EDs had higher scores in EOT, a facet of alexithymia and hence emotion processing; in relation to emotion regulation, they reported more difficulties accessing emotion regulation strategies, and more impulse control difficulties compared to females with EDs. They also reported using more reappraisal and suppression than did women with EDs. Note that while higher scores in most of these variables are indicative of greater impairment or maladaptive emotion regulation strategies, higher scores in reappraisal suggest more frequent use of this adaptive strategy.

The main effect of sex on emotion processing and emotion regulation still held after controlling for general distress, *F*(11, 274) = 4.34, *p* < 0.001, Wilk’s Λ = 0.852, partial η^2^ = 0.15), although the only statistically significant differences between groups were found for EOT, reappraisal and suppression.

### Part 3: does sex moderate the relationship between emotion difficulties and eating psychopathology?

The model significantly predicted general eating psychopathology in the whole sample, *F*(23, 1569) = 71.9, *p* < 0.001, adj. *R*^2^ = 0.35). Significant predictors of eating psychopathology were sex (*b* = 0.59, *p* < 0.001), limited access to emotion regulation strategies (*b* = 0.08, *p* < 0.001), non-acceptance of emotions (*b* = 0.8, *p* < 0.001), and impulse control difficulties (*b* = 0.06, *p* < 0.001). The main effect of sex in the absence of interaction suggests that being female was linked to higher eating psychopathology, but that the association between emotion processing and regulation and general eating psychopathology was not different between the two sexes.

When looking at specific aspects of eating psychopathology with the EDEQ subscales, we found that the model also predicted shape concerns (*F*(23, 1569) = 37.6, *p* < 0.001, adj. *R*^*2*^ = 0.35); weight concerns, *F*(23, 1569) = 36.2, *p* < 0.001, adj. *R*^*2*^ = 0.36); and eating concerns, *F*(23, 1569) = 37.1, *p* < 0.001, adj*. R*^*2*^ = 0.34). For all three EDEQ subscales above, we found significant effects of sex that reflected a general tendency for women to exhibit greater shape concerns (*b* = 0.71, *p* < 0.001), greater weight concerns (*b* = 0.69, *p* < 0.001), and greater eating concerns (*b* = 0.37, *p* < 0.001). In relation to emotion processing and regulation variables, predictors significant in all three models for EDEQ subscales included limited access to emotion regulation strategies (*b* = 0.11, *p* < 0.001 for shape concerns, *b* = 0.09, *p* < 0.001 for weight concerns, *b* = 0.08, *p* < 0.001 for eating concerns), non-acceptance of emotions (*b* = 0.09, *p* < 0.001 for shape concerns, *b* = 0.10, *p* < 0.001 for weight concerns, *b* = 0.08, *p* < 0.001 for eating concerns), and impulse control difficulties (*b* = 0.06, *p* < 0.001 for shape concerns, *b* = 0.07, *p* < 0.001 for weight concerns, *b* = 0.07, *p* < 0.001 for eating concerns). Only in the model for eating concerns were additional significant contributions made by difficulty identifying emotions (*b* = 0.03, *p* < 0.001) and reappraisal (*b* = − 0.02, *p* < 0.001).

**Fig. 1:**
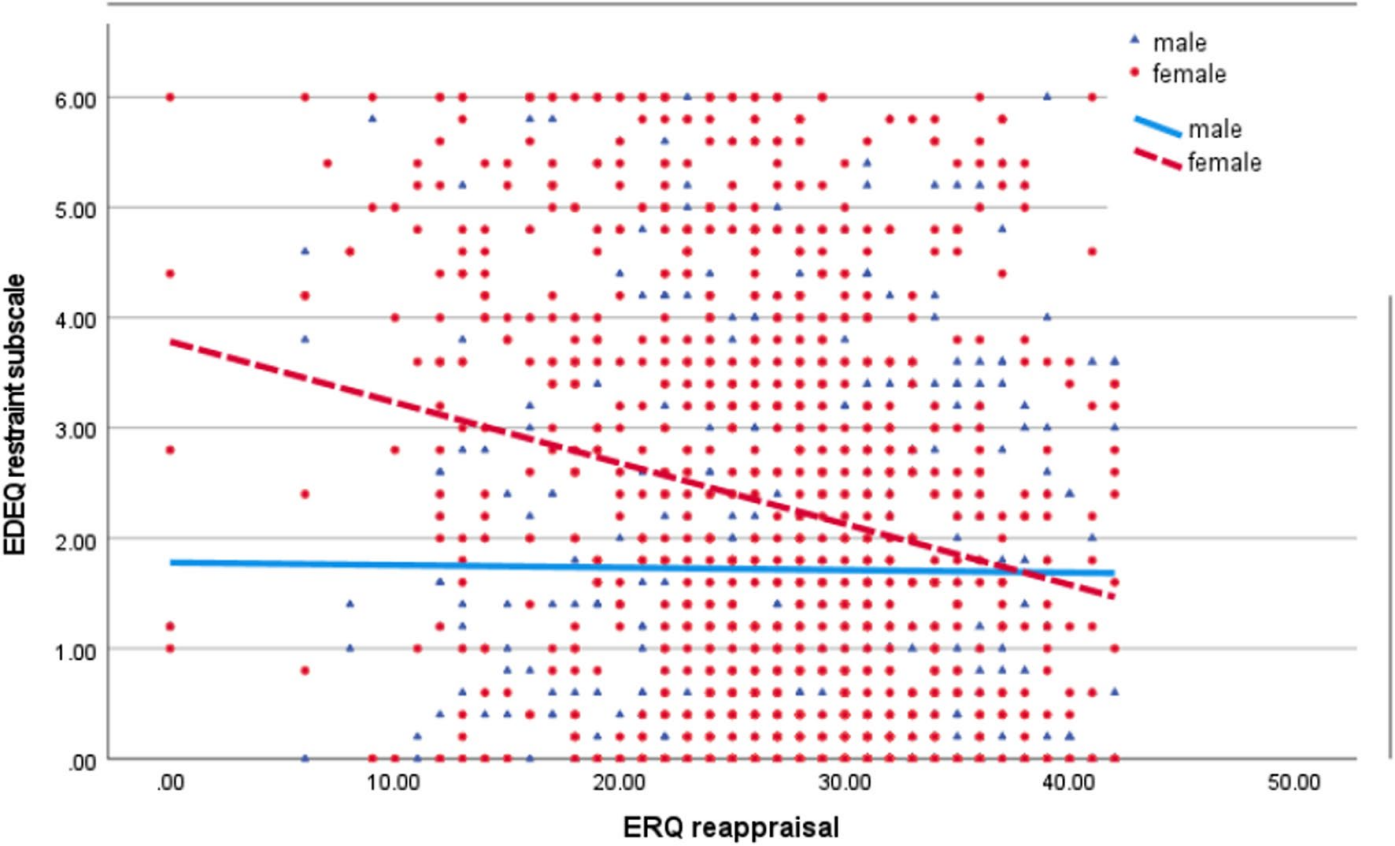
Interaction sex by reappraisal on the EDEQ restraint subscale

While models for shape, weight and eating concerns were highly similar, the model for restraint differed somewhat (*F*(23, 1569) = 15.0, *p* < 0.001, adj. *R*^*2*^ = 0.17). Women reported more eating restraint than men (*b* = 0.48, *p* < 0.001), and emotion-related predictors in this model included non-acceptance of emotions (*b* = 0.07, *p* < 0.001) and impulse control difficulties (*b* = 0.06, *p* < 0.001). The main difference with the other models was that we found a sex by reappraisal interaction (*b* = − 0.05, *p* < 0.001). As represented in Fig. 1, this interaction suggests that the use of reappraisal was unrelated to restraint in males, but that lower use of reappraisal was associated with higher levels of restraint. Note that because this section was an exploratory analysis, we ran an additional post-hoc analysis with only reappraisal as an emotion predictor on all EDEQ outcomes. This analysis showed a significant sex by reappraisal interaction for all scales of the EDEQ: the total scale (b = − 0.15, *p* < 0.001), eating concern (b = − 0.03, *p* < 0.001), shape concern (b = − 0.12, *p* = 0.002) and weight concern (b = − 0.13, *p* = 0.001), suggesting that the association between reappraisal use and eating psychopathology is stronger in females than males for all aspect of eating psychopathology, but that it was probably not significant due to other significant predictors explaining more of the variance in the models with all emotion predictors.

Each individual participant is represented by a dot or triangle (red dot for females, blue triangle for males). The lines represent the regression slope for each group (dotted red for females and bold blue for males).

All analyses were replicated to control for general distress and results remained mainly identical, including the sex by reappraisal interaction that was found for both restraint and eating concern, suggesting that it may be specific to eating psychopathology, rather than due to general distress associated with EDs (See Additional file 1).

## Discussion

This study aimed to assess the presence and relevance of emotion processing and emotion regulation difficulties in males and females with EDs. While difficulties with emotion processing and regulation are believed by some to constitute a transdiagnostic and important feature of EDs in women [21, 60, 61], men with EDs have largely been excluded from this body of work [25]. In a large sample of 1604 participants (of whom 631 were male), we contrasted the emotional functioning of men with EDs firstly in relation to healthy male controls, and secondly to female counterparts with EDs. Just like their female counterparts, men with EDs showed impairments in emotion processing and emotion regulation in comparison to healthy males without EDs. However, the comparison of men and women with EDs revealed differences between these groups. Men with EDs reported using more reappraisal and more suppression, as well as displaying a more externally-orientated thinking style than females with EDs. They also reported more difficulties accessing emotion regulation strategies and more impulse control difficulties than females with EDs, although differences in these variables were no longer statistically significant after controlling for general distress. Importantly, the third goal of our research concerned the relevance of these difficulties for ED psychopathology. We found that difficulties with emotion processing and regulation predicted a wide range of eating psychopathology in both males and females. While difficulties in these areas thus appear relevant to ED psychopathology in men and women alike, we also found a significant moderating effect of sex on the relationship between the use of reappraisal and restraint symptoms: this effect suggested that the use of reappraisal was not significantly associated with restraint in men, while lower use of this strategy was predictive of restricted eating in women. We will discuss the findings related to our three aims in turn before exploring the limitations and implications of our study.

### Profiling emotion processing and emotion regulation in men with ED against their relevant counterparts

Emotion processing and regulation difficulties have been robustly recognised in EDs and indeed conceptualised as a major causal and transdiagnostic feature [21, 60, 61, 78]. That these deficits should be present in men, however, has generally been only *assumed*, given that this conceptualisation of EDs is based predominantly on empirical findings that do not represent men or people with minority sexual or gender identities [25, 79]. The present study corroborates a very small literature supporting the presence of emotion processing and regulation deficits in men with EDs [7]. Our findings confirmed the presence of emotion regulation deficits in five of the six subscales, namely Strategies, Non-acceptance, Impulse, Goals and Clarity subscales. These findings extended those of Agüera and colleagues [7] in two important ways. Firstly, we were able to confirm that difficulties with accessing strategies, accepting emotions, and controlling impulses when upset were *independent* of general distress. Furthermore, our study used a more extensive battery of measures which are believed to more precisely delineate early- and late-stage emotion deficits. As such, with these measures, we were able to observe difficulties identifying and describing emotions, and an externally-orientated thinking style in men with EDs. It is however important to note that the EOT scale had lower internal consistency in our sample (Cronbach’s alpha below.70), and while this is common for this scale [80], our results should be treated with caution. Men with EDs also reported using more suppression than HC men. These findings are important in that they indicate that men with EDs show differences over and above the general tendency for men to exhibit higher levels of alexithymia [32, 33]. “Socially-encultured alexithymia” in men has been linked to Western norms around masculine identity [32, 33], but our findings suggest that men with EDs stand out from their male peers in much the same way as women with EDs stand out from HC women.

Our second contrast of interest was between men and women with EDs, a comparison which aimed to reveal whether emotion processing and regulation deficits are uniform across people with EDs or differ in accordance with sex. Interestingly, although men with EDs appeared to have more difficulties accessing strategies and inhibiting impulses when upset, controlling for general distress rendered these differences non-significant, suggesting that in relation to the DERS, men and women with EDs were more similar than different. These findings were not entirely comparable with those of Agüera et al. [7], who found that their *female* sample scored more highly than their males on these two DERS subscales (Strategies, Impulse). The difference may lie in the form of the DERS used (short vs. full) or, more likely, the fact that these authors did not control for general distress, or even length or severity of illness. We felt it was necessary to control for length or severity of illness because these differed between our male and female participants, and we wanted to look at *sex* differences, rather than differences due to the severity of the illness.^2^ Beyond the DERS, however, we did find some sex differences distinct from general distress: men were more likely to suppress their emotions; more likely to use reappraisal; and more likely to exhibit externally-orientated thinking. These sex differences in relation to suppression and externally-orientated thinking are consistent with male norms [31, 33–36]. Sex differences in reappraisal are less consistent in the general public [40], but there is some suggestion that men may generally find this process less effortful [81], and difficulties with reappraisal have been highlighted as a particular difficulty for women with EDs in comparison with HC women [82]. Caution must be exercised though, given that self-evaluation in groups with high perfectionism and low self-esteem, such as individuals with EDs [83, 84], may be an inaccurate reflection of real behaviour. Because men tend to report higher self-esteem than females [85], it is possible that males and females do not differ in their actual use of reappraisal, but females just report it less due to self-esteem issues. As we did not measure perfectionism or self-esteem and did not assess the validity of self-evaluations, this interpretation is possible. Issues of self-evaluation aside, the clinical relevance and importance of this sex difference can only be ascertained in relation to our third research goal, to which we now turn.

### Differential relevance of emotion processing and regulation deficits to ED psychopathy in men and women

Observed emotion processing and regulation difficulties in EDs, and the sex differences that characterise male and female EDs, are important only in so far as they are *relevant* to the pathogenesis and maintenance of EDs, and relatedly, the extent that interventions targeting these difficulties might ameliorate ED symptomatology. There is, as mentioned, extant literature purporting a central role for emotion processing and regulation deficits in the ED spectrum [21, 53, 60, 86], albeit in *female* samples, and these difficulties are hence addressed in psychological therapies for EDs. There is also preliminary support for the beneficial impact of emotion processing- and emotion regulation-focused interventions on ED symptomatology [80, 87]. Against the backdrop of this literature, our findings support the perspective that emotion processing and regulation deficits are important in the aetiology of EDs. Here, again, men were more similar than dissimilar to women. In both groups, emotion regulation difficulties most predictive of ED psychopathology were non-acceptance of emotion and difficulties controlling impulses when upset, limited access to emotion regulation strategies, and reappraisal. Only one facet of alexithymia—difficulty identifying emotions—was associated with eating concerns. This facet is conceptualised to manifest early in the emotion cycle and has been particularly linked with EDs [74, 75, 88] and with broader emotion regulation difficulties, somatic complaints and psychopathology [9, 89]. While multicollinearity was not problematic with our independent variables, the DERS and TAS-20 measures are known to share variance [16, 44], which may have limited our ability to fully delineate effects between these variables. Nevertheless, the relevance of these emotion processing and regulation differences to ED psychopathology in men (as in women) is an important redress to the under-representation of these individuals in ED research, and is consistent with parallel but plausibly related findings which implicate emotion regulation difficulties, in men, with muscle dysphoria and pathological pursuit of muscularity [90].

Interestingly, the greater difficulties seen in men with EDs as compared to women with EDs—namely greater alexithymia, more difficulties accessing emotion regulation strategies and controlling impulses, and greater use of suppression – did not translate to greater relevance of these factors for EDs in males (as might have been reflected by more severe symptoms). The single moderating effect of sex concerned the importance of reappraisal for restraint symptoms: while the use of this strategy was unrelated to the severity of restraint in men, lower use of this strategy was associated with higher levels of restraint symptoms in women. Our post-hoc analysis involving only reappraisal even suggested that this moderation effect might also be pertinent to the other facets of the EDEQ as well as total EDEQ score. It also does not seem to be an isolated finding as the same pattern has been found before in relation to depression, such that in females, using reappraisal seems associated with fewer depressive symptoms compared to males [91]. While this is an interesting finding, it requires further investigation with designs capable of clarifying causal or directional relationships between variables. As the present cross-sectional study cannot do this, two hypotheses are plausible: either using reappraisal may *protect* women against ED psychopathology (which would explain why our women reporting high reappraisal also reported lower psychopathology), or it could also be that women with high eating psychopathology do not find reappraisal useful and hence are less inclined to attempt it (which would explain why our females with a high level of psychopathology also reported lower reappraisal). Both hypotheses find support in the literature. The fact that reappraisal may be helpful against eating disordered behaviours is supported by recent studies such as one by Fitzpatrick et al. [58], who found that training women with EDs to use reappraisal resulted in reductions in body dissatisfaction and shape-based self-esteem schemas, although it did not reduce the perceived likelihood of engaging in ED behaviours. However, the hypothesis that women with high levels of ED behaviour may be less able to use reappraisal also has strong support from the literature. Indeed, the efficacy of cognitive reappraisal varies across contexts [92, 93], with people generally tending to reserve this strategy for situations of low emotional intensity [92], presumably because it is too difficult or ineffective to use reappraisal when one is overwhelmed with emotions. We also know that people with EDs tend to experience emotions with high intensity, partly due to alexithymia [5, 9], so it is possible that females with high levels of ED behaviours do not use reappraisal because they get more easily overwhelmed with emotions due to alexithymia, rendering reappraisal ineffective. However, what remains unclear is why our male sample does not seem affected by this. Our male and female samples did not differ in reported alexithymia, but it is possible that they differed in their experiences of emotional intensity [94, 95] which we did not measure, or it could be due to the fact that they rely on other emotion regulation strategies not measured here. Another explanation could be due to increased cognitive rigidity, which is a known side effect of starvation [96, 97]. Our men with EDs scored lower than our females on the restraint eating subscale, so it is possible that they were less susceptible to the physical effects of starvation and perhaps less cognitively rigid and more able to use reappraisal, which requires cognitive flexibility. These hypotheses are speculative at present, as the present study did not afford means to test them. Indeed, we did not measure BMI given the diversity of EDs in our participants, as BMI is an inadequate representation of individual malnutrition relative to that person’s own optimal state and hence not a reliable indicator of malnutrition-induced cognitive rigidity. Ultimately, while future studies should investigate the directionality of our finding, the fact that reappraisal did not affect eating psychopathology in males shows that we must be careful when implementing interventions that target a specific emotion regulation strategy, as it may not be effective for all people affected by EDs. The validity of theories of causal and maintaining factors in EDs, and subsequent psychological interventions based on the same, must therefore be tested in minority groups.

## Limitations and future directions

While our study is the first to date to compare males and females with EDs on a range of emotion regulation strategies as well as alexithymia, it is not without limitations. Principally, it is important to recognise that the cross-sectional nature of our study disallows any inferences of directionality between variables. This is especially important in case emotion processing and regulation difficulties are *consequential* to ED psychopathology as opposed to causal factors in its aetiology. While some studies suggest the malnourishment associated with EDs can exacerbate alexithymia [98] and affect the presentation of emotional dysregulation [99], other studies support the causal primacy of emotion processing and regulation deficits over ED psychopathology [22] and, as far as AN is concerned, the persistence of difficulties in these areas after weight-restoration [100, 101]. Compelling evidence for this perspective comes from Racine and Wildes [102], whose longitudinal findings suggested emotion regulation difficulties were independent of BMI and predictive of changes in the severity of AN symptoms, while the reverse relationship (AN severity predicting emotion regulation difficulties) was unsupported. Nevertheless, our regressions cannot ascribe causal primacy of our emotion processing and regulation variables over ED psychopathology.

Other factors which could potentially have confounded our group comparisons included the time at which the data was collected. Our complete dataset came from two datasets collected at two different time points: before and during the pandemic, with the majority of our ED sample (79.8%) having been collected during the pandemic. Because we know that the pandemic aggravated ED psychopathology [19], this could have inflated the differences between our ED vs non-ED group. However, because the majority of our ED group was collected during the same period, we believe that it did not influence the sex differences in emotion processing and regulation found *within* this group. Another issue with our sample lies in the fact that we relied on self-reports of ED diagnoses, lacking the time and resources to clinically assess all 329 participants with an ED. This means we cannot ascertain their diagnoses, and we recommend future studies to replicate our findings with independently validated diagnoses. Also, while our sample size is the largest of any previous attempts to examine sex differences in emotion processing and regulation within ED populations, we did not have the power to compare men and women with each subtype of ED. While some studies report highly similar use of maladaptive and adaptive emotion regulation strategies across ED subtypes [4], others point out differences in the use of adaptive emotion regulation strategies specifically [18]. Future research should investigate such potential differences in more targeted statistical investigations.

Finally, it is important to consider the representativeness and generalisability of our sample. First, our participants with ED were recruited from the community rather than specialised services, and while they may better represent the general population with an ED, particularly males with ED (e.g., 23), our results may not be comparable with other studies examining participants receiving treatment. Additionally, our sample was predominantly white British (60.5%). As EDs present differently in different cultures [103], and because culture also influences sex differences in emotion regulation [104], future research must consider this vital factor in relation to emotion processing and emotion regulation within ED populations of different sexes. Relatedly, although cisgender men are certainly a minority group whose difficulties and needs require attention within ED research and services, our study did not consider the presentation or relevance of emotion processing and regulation difficulties in other sexual and gender minorities within the ED population. Being likewise under-studied, it is important to test assumptions about psychopathological factors in the aetiology and maintenance of EDs in these individuals, given that difficulties targeted by interventions could be more or less relevant in different groups.

## Conclusion

We found that difficulties with emotion processing and emotion regulation were associated with eating psychopathology in both males and females. We also found that in comparison to women with ED, men with ED reported more externally-orientated thinking (a facet of alexithymia), more difficulties with their emotions, and more use of reappraisal and suppression of emotions. Differences with emotion processing and emotion regulation variables were predictive of ED psychopathology in men *and* women, suggesting that more difficulties with emotions were not associated with more eating difficulties in males. Interestingly, however, sex was found to moderate the relationship between reappraisal and restraint: the extent to which male participants used reappraisal was unrelated to this symptom, while lower use of reappraisal was associated with greater restraint in women. This suggests we need to follow a cautious approach when implementing interventions targeting reappraisal in EDs, as they may not be applicable to everyone. Moderating the effects of sex and gender on causal factors and treatment responses should be a crucial target for future research in order to advance inclusivity in this field.

## Supplementary Information

**Additional file 1: Supplementary material 1:** Regression models for all subscales of the EDEQ, controlling for general distress.

## Data Availability

The datasets used and/or analysed during the current study are available from the corresponding author on reasonable request.

## Abbreviations

AN: Anorexia nervosa
BED: Binge eating disorder
BN: Bulimia nervosa
DASS: Depression anxiety stress scale
DERS: Difficulties in emotion regulation scale
DV: Dependent variable
ED: Eating disorder
EDEQ: Eating disorder examination questionnaire
EDNOS: Eating disorder not otherwise specified
ERQ: Emotion regulation questionnaire
IV: Independent variable
OSFED: Other specified feeding or eating disorder
TAS: Toronto alexithymia scale

## Footnotes

1 Unfortunately, scientific research has traditionally neglected to differentiate between sex and gender identity. While we recognise the separability of these two aspects of identity and the importance of this distinction in psychopathology (see, for e.g., Hartung and Lefler [27]), our use of ‘men’ and ‘women’, ‘girls’ and ‘boys’ should henceforth be assumed to reflect cisgender people unless otherwise specified.

2 While we felt that the difference between groups necessitated controlling for length or severity of illness, we recognise that this may have removed relevant variance from the analysis if EP and ER are indeed central to ED psychopathology and hence naturally associated with more severe illness. Future research may avoid this conundrum by recruiting more equally matched samples with both acute and prolonged presentations.
