# Supplementary material for: What about males? Exploring sex differences in the relationship between emotion difficulties and eating disorders
Source: J Eat Disord. 2022 Dec 13;10:193. doi: 10.1186/s40337-022-00715-6 (PMC9749243; doi:10.1186/s40337-022-00715-6)
Supplement: Supplementary file 1 — Additional file 1: Supplementary material 1: Regression models for all subscales of the EDEQ, controlling for general distress. [file 40337_2022_715_MOESM1_ESM.docx]

**Supplementary material**

**Supplementary material 1: Regression models for all subscales of the EDEQ, controlling for general distress.**

|  | **EDEQ total** | | **Shape concerns** | | **Weight concerns** | | **Eating concerns** | | **Restrictive eating** | |
| --- | --- | --- | --- | --- | --- | --- | --- | --- | --- | --- |
|  | *F*(13, 1578)=43.6, *p*<.001, adj. *R*^2^=.39) | | *F*(13, 1578)=41.8, *p*<.001, adj. *R*^2^=.38) | | *F*(13, 1578)=41.4, *p*<.001, adj. *R*^2^=.38) | | *F*(13, 1578)=42.9, *p*<.001, adj. *R*^2^=.39) | | *F*(13, 1569)=16.5, *p*<.001, adj. *R*^2^=.19) | |
|  | ***b*** | ***p*** | ***b*** | ***p*** | ***b*** | ***p*** | ***b*** | ***p*** | ***b*** | ***p*** |
| Sex | .61 | <.001* | .73 | <.001* | .71 | <.001* | .39 | <.001* | .49 | <.001* |
| DASS | .02 | <.001* | .02 | <.001* | .02 | <.001* | .02 | <.001* | .02 | <.001* |
| DIF | -.00 | .721 | -.01 | .438 | -.01 | .681 | .00 | .941 | .00 | .848 |
| DDF | .00 | .759 | .02 | .236 | .01 | .652 | .01 | .577 | -.02 | .139 |
| EOT | .02 | .115 | .01 | .234 | .02 | .137 | .01 | .137 | .02 | .166 |
| Strat | .06 | .032* | .08 | .011 | .05 | .075 | .05 | .057 | .03 | .337 |
| NA | .04 | .066 | .04 | .110 | .06 | .019 | .04 | .125 | .03 | .241 |
| Imp | .07 | .003* | .06 | .017 | .07 | .008 | .10 | <.001* | .05 | .056 |
| Goals | .01 | .678 | .04 | .118 | -.00 | .966 | -.02 | .488 | -.01 | .755 |
| Awa | -.02 | .294 | -.01 | .696 | -.02 | .334 | -.02 | .316 | -.03 | .223 |
| Clar | -.00 | .910 | -.02 | .427 | -.01 | .863 | -.02 | .499 | .04 | .191 |
| Reap | .01 | .145 | .01 | .321 | .01 | .172 | .00 | .833 | .03 | .012 |
| Sup | -.01 | .338 | -.01 | .737 | -.01 | .388 | -.00 | .750 | -.03 | .044 |
| Sex * DIF | .00 | .868 | .00 | .911 | .00 | .913 | .01 | .696 | .00 | .881 |
| Sex * DDF | -.02 | .429 | -.03 | .239 | -.02 | .263 | -.02 | .439 | .00 | .853 |
| Sex * EOT | -.01 | .739 | -.00 | .861 | .00 | .880 | -.01 | .536 | -.01 | .737 |
| Sex * Strat | -.01 | .754 | -.01 | .812 | .00 | .930 | -.01 | .755 | -.02 | .615 |
| Sex * NA | .03 | .239 | .04 | .197 | .03 | .398 | .03 | .266 | .03 | .454 |
| Sex * Imp | -.03 | .290 | -.03 | .418 | -.02 | .523 | -.08 | .007* | -.01 | .842 |
| Sex * Goals | -.04 | .176 | -.05 | .107 | -.03 | .313 | .00 | .998 | -.06 | .103 |
| Sex * Awa | .03 | .344 | .03 | .434 | .04 | .261 | .02 | .605 | .02 | .598 |
| Sex * Clar | .03 | .382 | .06 | .148 | .03 | .517 | .04 | .282 | -.01 | .892 |
| Sex * Reap | -.03 | .013* | -.02 | .160 | -.02 | .095 | -.03 | .011* | -.05 | <.001* |
| Sex * Sup | .01 | .041* | .01 | .801 | .01 | .538 | .00 | .827 | .04 | .062 |

The predictors are Sex; General distress (DASS-21 total score); the three scubscales of the Toronto Alexithymia Scale (TAS-20), namely DIF (Difficulties Identifying Emotions), DDF (Difficulties Describing Emotions), and EOT (External Oriented Thinking); the six subscales of the Difficulties in Emotion Regulation Scale (DERS), namely Strat (Limited Access to Emotion Regulation Strategies), NA (Non Acceptance of emotions), Imp (Impulse Control Difficulties) Goals (Difficulties Engaging in Goal-Directed actions), Awa (Lack of Emotional Awareness), and Clar (Lack of Emotional Clarity); the two subscales of the Emotion Regulation Questionnaire (ERQ), namely Reap (Reappraisal) and Sup (Suppression); finally, predictors in the model include 11 interaction terms reflecting sex by these emotion variables interactions. Significant effects are denoted with * indicating p < .05.
